# Evaluating bat boxes: design and placement alter bioenergetic costs and overheating risk

**DOI:** 10.1093/conphys/coac027

**Published:** 2022-04-25

**Authors:** Reed D Crawford, Luke E Dodd, Francis E Tillman, Joy M O’Keefe

**Affiliations:** Department of Biological Sciences, Eastern Kentucky University, Kentucky 40475, USA; Program in Ecology, Evolution, and Conservation Biology, University of Illinois at Urbana-Champaign, Illinois 61801, USA; Department of Biological Sciences, Eastern Kentucky University, Kentucky 40475, USA; Center for Bat Research, Outreach, and Conservation, Indiana State University, Indiana 47809, USA; Department of Biological Sciences, The University of Memphis, Tennessee 38152, USA; Program in Ecology, Evolution, and Conservation Biology, University of Illinois at Urbana-Champaign, Illinois 61801, USA; Department of Natural Resources and Environmental Sciences, University of Illinois at Urbana-Champaign, Illinois 61801, USA; Center for Bat Research, Outreach, and Conservation, Indiana State University, Indiana 47809, USA

## Abstract

Bat box microclimates vary spatially and temporally in temperature suitability. This heterogeneity subjects roosting bats to a variety of thermoregulatory challenges (e.g. heat and cold stress). Understanding how different bat box designs, landscape placements, weather and bat use affect temperature suitability and energy expenditure is critical to promote safe and beneficial artificial roosting habitat for species of conservation concern. From April to September 2019, we systematically deployed 480 temperature dataloggers among 40 rocket box style bat boxes of 5 designs and regularly monitored bat abundance. We used bioenergetic models to assess energy costs for endothermic and heterothermic bats and modelled the overheating risk for each box as a function of design, placement, bat abundance and weather. For endothermic bats, predicted daily energy expenditure was lower for solar-exposed placements, large group sizes and a box design with enhanced thermal mass. For heterothermic bats, shaded landscape placements were the most energetically beneficial and bat box design was not important, because all designs generally offered microclimates suitable for torpor use at some position within the box. Overheating risk was highest for solar-exposed landscape placements and for designs lacking modifications to buffer temperature, and with increasing bat abundance, increasing ambient temperature and slower wind speeds. The external water jacket design, with the greatest thermal mass, concomitantly decreased overheating risk and endothermic energy expenditure. By assessing bat box suitability from two physiological perspectives, we provide a robust method to assess the conservation value of bat box design and placement strategies. We recommend future studies examine how changing thermal mass and conductance can be used to diminish overheating risk while also enhancing the effects of social thermoregulation for bat box users.

## Teaser

We assess bioenergetic costs and overheating risk of five artificial roost designs. For endothermic bats, energy expenditure was lower for solar-exposed locations and our enhanced thermal mass box design. Shaded landscape placements reduced energy expenditure for heterothermic bats. Increasing ventilation, surface reflectance, thermal mass and decreasing solar exposure decreased overheating.

## Introduction

Anthropogenic land-use change has decreased natural habitats for bats worldwide (Frick *et al.*, 2020). To offset roost habitat loss or enhance landscapes, practitioners often turn to bat boxes (Flaquer *et al.*, 2006; Whitaker *et al.*, 2006). Recently, however, the efficacy of bat boxes has been called into question (Flaquer *et al.,* 2014; Griffiths *et al.,* 2017). While provisioning energetically beneficial bat boxes could help imperilled species recover from diseases like white-nose syndrome (Webber and Willis, 2018; Wilcox and Willis, 2016), many bat boxes are inadequate at buffering extreme temperatures (e.g. Flaquer *et al.,* 2014; Griffiths *et al.,* 2017; Hoeh *et al.*, 2018; Martin Bideguren *et al.,* 2019; Rueegger, 2019). Additionally, bats avoid some designs and suboptimal landscape placements, which leads to limited conservation benefit (Rueegger *et al.*, 2019; Whitaker *et al.*, 2006). Identifying design and placement combinations supporting beneficial microclimates and promoting occupancy could enhance the efficacy of bat boxes as conservation tools by reducing overheating events (Martin Bideguren *et al.,* 2019), promoting energy savings (Wilcox and Willis, 2016), facilitating disease recovery (Fuller *et al.,* 2020) and enhancing pup development (Zahn, 1999).

For bats, finding roosts that suit their thermoregulatory needs is vital to survival and pup development during pregnancy and lactation. Due to high surface area to volume ratios and low body mass, bats often thermoconform to roost temperature (Czenze *et al.*, 2020; Licht and Leitner, 1967a). Warm roosting conditions enhance pup development because less energy is spent on maintaining endothermy (i.e. maintenance of stable core body temperature through metabolic heat production; Zahn, 1999; Lausen and Barclay, 2006). Furthermore, maintaining endothermy in cold roosts can be energetically expensive (Geiser and Brigham, 2000; Willis *et al.,* 2005a). Consequently, many reproductively active individuals will use torpor (i.e. become heterothermic) when roosts are cool (Bergeson *et al.*, 2021; Willis *et al.*, 2006), even though this behaviour involves reproductive costs of delayed parturition (Racey and Swift, 1981), reduced milk production (Wilde *et al.*, 1999) and slowed juvenile development (Hoying and Kunz, 1998).

Roost temperatures occasionally exceed bats’ heat tolerance limits (Flaquer *et al.,* 2014; Griffiths, 2021; Griffiths *et al.,* 2017; Martin Bideguren *et al.,* 2019). In the absence of temperature refugia within a roost, bats have limited capacity to combat lethally high temperatures but may respond by shifting location (Licht and Leitner, 1967b; Lourenço and Palmeirim, 2004), increasing evaporative cooling (Czenze *et al.*, 2020) and vasodilation of blood vessels (Reeder and Cowles, 1951) or using facultative hyperthermia (Reher and Dausmann, 2021). Temperatures exceeding 40°C may induce heat stress, which increases energy costs; prolonged exposure to high temperatures can cause mortality (Licht and Leitner, 1967a). If bats preferentially select roosts prone to temperature extremes and that offer no refugia, the roosts may function as ecological traps (Crawford and O’Keefe, 2021a).

Bat box microclimates are influenced by various abiotic factors, for example, by structural components like construction material (Martin Bideguren *et al.,* 2019; Rueegger, 2019), colour (Doty *et al.*, 2016; Griffiths *et al.,* 2017) and volume (Tillman *et al.*, 2021). For example, black three-chamber boxes average 5°C warmer than white three-chamber boxes (Lourenço and Palmeirim, 2004). Furthermore, environmental factors like cloud cover (Hoeh *et al.*, 2018), ambient temperature (Bartonička and Řehák, 2007), wind (Tillman *et al.*, 2021), humidity (Rueegger, 2019) and solar exposure (Brittingham and Williams, 2000) all modulate microclimate. For instance, on days with clear skies, bat box temperature ranges can vary by as much as 10°C from top to bottom, whereas on cloudy days there is little variation in box temperature top to bottom (Hoeh *et al.*, 2018). Finally, roost landscape placement will determine aspect (Mering and Chambers, 2012) and canopy shading (Kerth *et al.*, 2001), thus affecting box microclimate.

The physiological state of roosting bats may also impact bat box microclimate. For example, colony size and metabolic state (e.g. active or torpid) can influence box temperature and humidity (Bartonička and Řehák, 2007; Pretzlaff *et al.*, 2010). When in a torpid state, a bat’s body temperature is often near ambient conditions (Willis *et al.,* 2005a, 2005b); thus, individual torpid bats likely exert minimal influence on roost temperature. However, a large group of torpid bats adds thermal mass and insulation and, thus, should stabilize roost temperatures (Kurta, 1985). Conversely, socially thermoregulating bats (i.e. a group of normothermic individuals) could substantially increase roost temperature (Pretzlaff *et al.*, 2010), thereby yielding energy savings. For instance, the presence of bats in a roost could raise roost temperatures by 7°C, allowing group-roosting individuals to conserve ~ 53% of their daily energy budget (Willis and Brigham, 2007).

While bat boxes are deployed for bats worldwide, a lack of rigorous empirical research on microclimate and the effects of roosting bats has led to limited success and a poor understanding of bat boxes as conservation tools. Working at two study sites, we deployed 480 temperature dataloggers to map roost temperature at varying heights and aspects within 40 rocket-style bat boxes of 5 designs and assessed use by endangered Indiana bats (*Myotis sodalis*). For bats seeking to maintain endothermy, we expected solar-exposed locations, box designs promoting warmer temperatures, large bat group sizes and warm, calm weather would result in the lowest daily energy expenditure (DEE). For facultatively heterothermic bats, we expected shaded bat box placements, box designs promoting cooler temperatures, small group sizes and cold, windy weather would decrease DEE by reducing roost temperatures and, thus, facilitating deeper torpor. We hypothesized box designs with higher thermal mass, enhanced surface reflectance or enhanced ventilation would reduce overheating events compared to a reference box design. We further expected high solar exposure would increase overheating risk in all box designs.

## Materials and Methods

### Study sites

We conducted this study in Indiana and Kentucky, USA, from 1 April to 15 September 2019. The Indiana site, located in Hendricks County, included soybean, corn and wheat fields, grassy areas and restored wetlands, with small mixed forest fragments (about 10% of ~ 1045 ha area, Divoll and O’Keefe, 2018), and was bordered by urban housing and warehouse districts. During this study, the Indiana site accumulated 640 mm of total rainfall and outside air temperature (T_a_) ranged from 1.3°C to 34.7°C. Daily minimum and maximum temperatures averaged 13.5 ± 0.4°C and 26.7 ± 0.5°C, respectively. The Kentucky site, located in Scott County, was ~ 1010 ha and ~74% forested. The Kentucky site was characterized by mostly forested, rolling hills containing predominantly oak (*Quercus* spp.), hickory (*Carya* spp.) and eastern redcedar (*Juniperus virginiana*; Woods *et al.,* 2002). During the study, the Kentucky site accumulated 804 mm of total rainfall and T_a_ ranged from 2.5°C to 36.4°C. Daily minimum and maximum temperatures averaged 15.1 ± 0.4°C and 27.7 ± 0.4°C, respectively. Animal care and use protocols were approved by Eastern Kentucky University (no. 01-2019) and Indiana State University (no. 559972).

### Rocket box deployment

**Figure 1:**
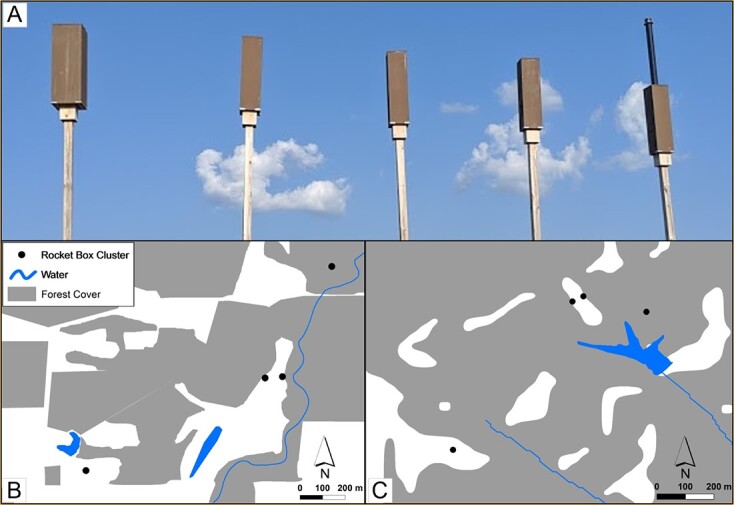
(A) Rocket box designs (from left to right): external water jacket (EJW), vent removal (VR), white tile roof (WTR), reference (REF) and chimney (CH). Locations of box clusters at the (B) Indiana and (C) Kentucky field sites.

We constructed eight replicates of five rocket box designs (i.e. 40 rocket boxes total; 20 per site). All design variants were modifications of the reference design described by Tillman *et al.* (2021). Our designs were reference (REF), vent removal (VR), chimney (CH), white tile roof (WTR) and external water jacket (EJW) (Fig. 1A). Design alterations were intended to promote microclimates different from the REF design while providing equal entrance area, roosting surface area and volume. Compared to REF, the VR design increases minimum temperatures by reducing ventilation, the CH design decreases maximum temperatures by venting rising hot air out a black chimney, the WTR design decreases maximum temperatures by reflecting solar radiation with a white ceramic tile roof and the EJW box buffers both high and low temperatures as a result of a sealed external chamber filled with three 750-ml water packets per box side, which increases thermal mass and, thus, time to heat and cool (Tillman *et al.*, 2021). We constructed boxes from 1.91-cm thick pine boards, offering a 1.91-cm chamber spacing, and coated exterior surfaces in two layers of medium brown paint. Seams were sealed with latex caulk to improve weather resistance.

At each site, we deployed four box clusters (hereafter, landscape placements; Fig. 1B and C), placing one of each of the five designs in each landscape placement to facilitate discovery and roost switching and provide bats a variety of microclimates in one locality (Lewis, 1995; Rueegger, 2016). Open solar treatment placements were located > 30 m away from tree lines and boxes received no shading. Forest placements were in a closed-canopy condition so boxes received little to no direct solar exposure. Easterly and westerly sun placements were ~5 m from east- and west-facing tree lines such that boxes primarily received morning and evening solar exposure, respectively. All boxes were deployed by 1 April 2019, which is earlier than the mean arrival date for Indiana bats at the Indiana site (3 April, Pettit and O’Keefe, 2017). Landscape placements ran along a north–south axis (box vents facing north and south), with box designs randomly ordered and spaced 2 m apart. The top of each rocket box was ~ 6 m above ground.

### Microclimate data collection

For all 40 boxes, we recorded internal air temperature (T_roost_) with Thermochron iButtons (Model DS1921G, Maxim Integrated, 0.5°C increments, ± 1.0°C accuracy). We placed iButtons at 12 identical positions in each box: top, middle and bottom on all four box faces, and only in the more variable outer chamber (Brittingham and Williams, 2000), as we were fundamentally interested in temperature extremes.

The iButtons were housed in plastic bushings that prevented bats from touching the iButton surface but did not affect temperature recordings and iButtons made no discernable ultrasonic noise when tested (Crawford, 2020). Our iButtons recorded temperature bi-hourly, on even or odd hour intervals to conserve memory space. Even and odd hour iButtons were alternated at each level within the box to ensure that temperature data were collected every hour at every level (i.e. top, middle, and bottom). We removed iButtons at the end of the study following 3 consecutive site visits with no bat detections (in October for both sites).

### Roost checks

To survey all 40 rocket boxes for presence/absence and abundance of Indiana bats, we performed spotlight checks and emergence counts 2–4 times per week per site, from April to October 2019 at both field sites. Spotlight checks, typically performed between 16:00–20:00 h (EDT), with sunset occurring around 21:00 h, involved shining a ~1000 lumen spotlight into each box and visually determining presence/absence of bats (Hoeh *et al.*, 2018; Whitaker *et al.*, 2006). Further, bats were visually counted to estimate abundance and to aid in determining where to conduct emergence counts. We classified bats to genus visually via spotlight checks; the only other bat species detected was the larger big brown bat (*Eptesicus fuscus*) on < 5% of observations and the presence of Indiana bats was reaffirmed via molecular diagnosis from faeces (Walker *et al.*, 2016) collected from guano traps beneath boxes. For emergence counts, observers arrived at boxes ~ 30 min before sunset and stayed 10 min after the last bat emerged or 30 min after sunset if no bats emerged (Hoeh *et al.*, 2018). Observers recorded times of first and last emergence and number of bats. Observers typically watched approximately three boxes within a box cluster. Observations of big brown bats were excluded from analyses.

### Weather data collection

We collected hourly weather data at each box cluster via Ambient Weather WS-1201 weather stations (four stations per site). Each weather station was mounted 3 m above ground and 2 m from the south side of each box cluster to prevent shading. Weather stations recorded temperature (°C), solar radiation (W/m^2^), rainfall (mm) and wind speed (m/s). Because recordings were hourly, we recognize that we may have missed some extreme weather observations. We discarded microclimate and bat count data for days when weather stations suffered power failures.

### Bioenergetic models

We used a bioenergetic modelling approach (following Humphries *et al.*, 2002, 2005; Wilcox and Willis, 2016), modified to assess the DEE of an Indiana bat that we assumed to use an optimal roosting position in each bat box over the study duration. By calculating DEE, we directly compare estimated energy expenditure of bats in each box design under different environmental conditions, while simultaneously accounting for the variety of temperatures available to exploit.

Because Indiana bats’ thermoregulatory behaviour can range from near perfect endothermy to extreme heterothermy (Bergeson *et al.*, 2021), we decided to model energy expenditures of two distinct thermoregulatory scenarios—continuous endothermy and facultative heterothermy. As detailed metabolic data are unavailable for the Indiana bat, we used data for the well-studied, morphologically similar little brown bat (*Myotis lucifugus)*. Using parameters listed in Table 1 and bioenergetic equations presented in Table 2, we calculated the DEE for a behaviourally thermoregulating bat occupying each bat box. For each scenario, we calculated mass-specific metabolic rate (mlO_2_g^−1^ hr^−1^) for a bat present in each bat box for a 24-h period. We then converted mass-specific metabolic rate to whole animal metabolic rate by multiplying by the mean body mass of reproductive female little brown bats (8.44 g; Kurta and Kunz, 1988). Then we converted hourly whole animal metabolic rates to energy expenditure in joules using a conversion factor of 1-ml O_2_ = 20.083 J (Schmidt-Nielsen, 1997). We summed the energy expenditure for each bat box for each day and converted joules to kilojoules for modelling.

**Table 1:** Parameters used in bioenergetic models to estimate the daily energy expenditure of roosting bats

**Parameter (Units)**	**Value**	**References**
BMR (mlO_2_g^−1^ hr^−1^)	2.6	Stones and Wiebers, 1967; Humphries *et al.*, 2002, 2005
T_lc_ (°C)	32	Hock, 1951; Stones and Wiebers, 1967; Humphries *et al.*, 2002, 2005
T_uc_ (°C)	36.26	Speakman and Thomas, 2003
C_eu_ (mlO_2_g^−1^ hr^−1^)	0.2638	Stones and Wiebers, 1967; Humphries *et al.*, 2002; 2005
T_onset_ (°C)	25	Geiser and Brigham, 2000; Cryan and Wolf, 2003; Willis *et al.,* 2005a; Geiser *et al.*, 2011
TMR_min_ (mlO_2_g^−1^ hr^−1^)	0.03	Hock, 1951; Humphries *et al.*, 2002, 2005
Q_10_	1.6 + 0.26T_roost_—0.006T_roost_^2^	Hock, 1951; Humphries *et al.*, 2002, 2005
T_tor-min_ (°C)	2	Hock, 1951; Humphries *et al.*, 2002, 2005
C_t_ (mlO_2_g^−1^ hr^−1^)	0.055	Hock, 1951; Humphries*et al.*, 2002, 2005
Mass (g)	8.44	Kurta and Kunz, 1988

The upper critical temperature, T_uc,_ is based on the mean value for 50 species provided in Speakman and Thomas (2003), all other values are specific to *Myotis lucifugus*. Parameters defined in text.

**Table 2:** Bioenergetic equations and criteria used to estimate the mass-specific metabolic rates of roosting bats in thermoregulatory Scenarios 1 and 2

**Scenario 1: Continuous Endothermy**	
**Criteria**	**Formula**
When T_roost_ ≥ T_lc_ and T_roost_ ≤ T_uc_	$\mathrm{BMR}$
When T_roost_ > T_uc_	$\mathrm{BMR}+({\mathrm{T}}_{\mathrm{roost}}\hbox{--} {\mathrm{T}}_{\mathrm{uc}})\ast {\mathrm{C}}_{\mathrm{eu}}$
When T_roost_ < T_lc_	$\mathrm{BMR}+({\mathrm{T}}_{\mathrm{lc}}\hbox{--} {\mathrm{T}}_{\mathrm{roost}})\ast {\mathrm{C}}_{\mathrm{eu}}$
**Scenario 2: Facultative Heterothermy**	
**Criteria**	**Formula**
When T_roost_ ≥ T_lc_ and T_roost_ ≤ T_uc_	$\mathrm{BMR}$
When T_roost_ > T_uc_	$\mathrm{BMR}+({\mathrm{T}}_{\mathrm{roost}}\hbox{--} {\mathrm{T}}_{\mathrm{uc}})\ast {\mathrm{C}}_{\mathrm{eu}}$
When T_roost_ < T_lc_ and T_roost_ > T_onset_	$\mathrm{BMR}+({\mathrm{T}}_{\mathrm{lc}}\hbox{--} {\mathrm{T}}_{\mathrm{roost}})\ast {\mathrm{C}}_{\mathrm{eu}}$
When T_roost_ ≤ 25°C and T_roost_ > T_tor-min_	${\mathrm{T}\mathrm{MR}}_{\mathrm{min}}\ast {\mathrm{Q}}_{10}^{({\mathrm{T}}_{\mathrm{roost}}\hbox{--} {\mathrm{T}}_{\mathrm{tor}-\min})/10}$
When T_roost_ ≤ T_tor-min_	${\mathrm{T}\mathrm{MR}}_{\mathrm{min}}+({\mathrm{T}}_{\mathrm{t}\mathrm{or}-\min}\hbox{--} {\mathrm{T}}_{\mathrm{roost}})\ast {\mathrm{C}}_{\mathrm{t}}$

Equations are based on those presented in Humphries *et al.* (2002, 2005). Parameters defined in text; values given in Table 1.

Because iButtons recorded every other hour, we knew temperatures at six roosting positions each hour (i.e. two at the top, middle and bottom of the box). In each thermoregulatory scenario, we assumed a behaviourally thermoregulating bat would select the roosting position that minimizes energy expenditure during each hour of a 24-h day; this assumption is logical as bats have been documented to shift along temperature gradients within a roost (Licht and Leitner, 1967b; Lourenço and Palmeirim, 2004). Thus, DEE values from each box for each day represent the lowest theoretical DEE for a bat using that specific thermoregulatory strategy.

Our first scenario modelled DEE for a continuously endothermic bat (DEE_endothermic_), such as a reproductively active female attempting to maintain high body temperature to facilitate pup development (Lausen and Barclay, 2006; Zahn, 1999). We assumed hourly roost temperatures within the thermoneutral zone result in metabolic costs equal to the basal metabolic rate (BMR; see Tables 1 and 2). When T_roost_ was less than the lower critical temperature (T_lc_), we assumed the energy expenditure was BMR plus the product of the temperature difference (between the critical temperature and roost temperature) and euthermic thermal conductance (C_eu_). Above the upper critical temperature (T_uc_), we assumed energy expenditure was BMR plus the product of the temperature difference and C_eu_, which increases linearly until lethal temperatures are reached. We note that some tropical bats may use hyperthermic torpor during heat stress (Reher and Dausmann, 2021), but this behaviour is currently documented for only a single bat species. Because we did not document any mortality during our study period (2019), we assumed bats could move to avoid experiencing lethal temperatures (i.e. ≥ 45°C).

Using the same microclimate data presented in scenario one, but applying a different set of equations, our second scenario modelled the DEE of a facultatively heterothermic bat selecting the temperature that minimizes energy expenditure each hour of the day (DEE_heterothermic_). This scenario assumes a reproductive or non-reproductive bat will attempt to maintain an endothermic body temperature until T_roost_ drops to the torpor onset value (T_onset_) when it will then enter torpor (see Tables 1 and 2). We chose T_onset_ = 25°C as several bat species readily enter torpor near this temperature (Geiser and Brigham, 2000; Cryan and Wolf, 2003; Willis *et al.,* 2005a; Geiser *et al.*, 2011). This scenario assumes bats will increase energy expenditure and attempt to maintain a warm body temperature when T_roost_ is below T_lc_ and above T_onset_ but once energy costs become too high (i.e. T_roost_ ≤ 25°C) bats will conserve energy through torpor. We concede that this cut off may over or underestimate energy expenditure based on the individual bats’ behaviour and physiological condition but is still a useful proxy for inference. While in torpor, metabolic rate is equal to the product of the minimum torpid metabolic rate (TMR_min_) and the temperature-dependent change in metabolic rate (Q_10_). If T_roost_ reaches the minimum torpid set point (T_tor-min_) of 2°C (noting the lowest temperature recorded in a box in this study was −7.5°C), the bat will increase energy expenditure to defend a minimum body temperature as a function of the temperature difference and torpid thermal conductance (C_t_), plus TMR_min_. Following Willis and Brigham (2007), we did not calculate costs of entry into and arousal from torpor, as the cost, duration and frequency of these events can be highly variable. After calculating DEE values, we paired these data with available bat counts and weather data (maximum daily temperature [°C] and windspeed [m/s]), yielding 2530 DEE estimates to analyse for each scenario.

Our DEE_heterothermic_ scenario assumes a bat moves among positions in the box, always choosing the position that minimizes DEE, even while torpid; however, we acknowledge that this is unlikely to happen in reality. Conservatively, we chose to model DEE using only roost positions with known temperature data. Because iButtons were recording every 2 h, this necessitated using different roosting positions in our calculations for hourly DEE. An alternate approach could assume the bat chooses the best position within the roost for torpor at the start of each day (assuming temperatures ≤ 25°C are available) and stays there until T_roost_ > T_onset_, thus allowing movement. One problem with this approach is that we would have to estimate T_roost_ for alternating hours when iButtons were not recording. Further, in our estimation, we could not account for the elevated temperatures at iButton positions within close proximity of groups or individual free-ranging bats that move non-randomly within roosts during the day. Our modelling approach for a facultative heterothermy scenario allows for a bat to move to the best roost location available for torpor and, thus, reduces this bias and models a clearer effect of box design itself on DEE.

### Overheating risk

In addition to measuring the metabolic costs associated with each box design, we investigated overheating risk. Here, we deemed temperatures > 40°C to be unsuitably hot, as bats avoid these high temperatures (Licht and Leitner, 1967b; Lourenço and Palmeirim, 2004), and prolonged exposure can result in heat stress and mortality (Alcalde *et al.*, 2017; Flaquer *et al.,* 2014; Griffiths, 2021). For each bat box for each day, we summed the total number of hourly overheating events (any hourly recording > 40°C) from the 144 recordings per box on each 24-h day. Because forest placements logged few overheating events (*n* = 25), models incorporating this landscape placement failed to converge; therefore, we removed forest placements from this analysis. We paired daily counts of overheating events with observations of bat abundance and weather, resulting in 1932 daily observations.

### Analysis

To model DEE under two scenarios and the daily count of overheating events, we followed an information theoretic approach (Burnham and Anderson, 2002), constructing an *a priori* candidate set of 14 models (Table 3). Models comprised combinations of box design, landscape placement, total number of bats, maximum daily T_a_, and maximum daily wind speed. We used linear models (LMs), using qq-plots and histograms of residuals to assess normality of DEE. We log transformed DEE_heterothermic_ to achieve normality. We used generalized LMs fit to a Poisson distribution to assess the daily count of overheating events. We checked models for overdispersion and goodness of fit via overdispersion parameters and *R*^2^ values. For all analyses, we checked for multicollinearity among predictors via variance inflation factor (VIF) tests; all predictors had VIFs < 2. The REF design and open landscape placements were used as reference levels during modelling, as all box designs are variants of the REF and high solar exposure is important for maternity colony formation. Although our dataset contained repeated measures within each of the 40 boxes, we did not include boxID as a random effect in models. We reason that under identical conditions each box design should function the same and, thus, yield the same results. As such, the differences between identical boxes in different landscape positions are due to environmental effects, not the boxes themselves.

**Table 3:** Candidate set of 14 *a priori* models used in modelling two daily energy expenditure scenarios and counts of overheating events

Model Name	Parameters
null	-
m2	Design
m3	Placement
m4	Design + Placement + Design:Placement
m5	Design + Placement + Total_Bats + Design:Total_Bats
m6	Design + Placement + Total_Bats + Design:Placement + Design:Total_Bats
m7	Design + Placement + Total_Bats + Max_Temp + Design:Placement + Design:Total_Bats + Design:Max_Temp
m8	Design + Placement + Total_Bats + Max_Temp + Design:Placement + Design:Total_Bats + Placement:Max_Temp
m9	Design + Placement + Total_Bats + Max_Temp + Max_Wind + Design:Placement + Design:Total_Bats + Design:Max_Temp + Design:Max_Wind
m10	Design + Placement + Total_Bats + Max_Temp + Max_Wind + Design:Placement + Design:Total_Bats + Placement:Max_Temp + Placement:Max_Wind
m11	Design + Placement + Max_Temp + Design:Placement + Design:Max_Temp
m12	Design + Placement + Max_Temp + Design:Placement + Placement:Max_Temp
m13	Design + Placement + Max_Temp + Max_Wind + Design:Placement + Design:Max_Temp + Design:Max_Wind
m14	Design + Placement + Max_Temp + Max_Wind + Design:Placement + Placement:Max_Temp + Placement:Max_Wind

All modelling was conducted in R version 3.6.2 (R Core Team, 2019). We ranked models via AIC*_C_* (Akaike’s Information Criterion corrected for small sample sizes) using the package ‘bbmle’ (Bolker, 2020) to identify the top overall model(s). We considered models to be competing if ∆AIC*_C_* was ≤ 2 from the top model. If competing models were present, we constructed a 90% confidence set for model averaging (Burnham and Anderson, 2002) using the package ‘MuMIn’ (Barton, 2020). To reduce the possibility of excluding biologically important parameters that were in top model(s), we identified informative parameters as those with 85% confidence intervals not overlapping 0 (Arnold, 2010). Means are presented as ¯ ± SE unless otherwise stated. Predicted means were obtained through the package ‘emmeans’ (Lenth, 2020).

## Results

### Continuous endothermy scenario

**Figure 2:**
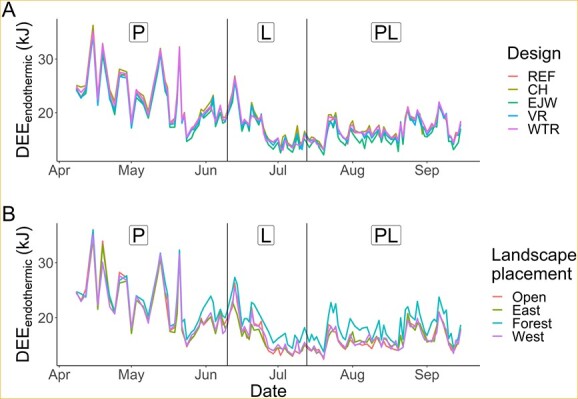
Mean daily energy expenditure for continuous endothermy (DEE_endothermic_) over the course of the study as a function of (A) box design: reference (REF), chimney (CH), external water jacket (EJW), vent removal (VR) and white tile roof (WTR), and (B) landscape placement: open sun (open), easterly sun (east), westerly sun (west), full shade (forest), recorded from 40 boxes divided among sites in Indiana and Kentucky. Vertical black lines designate reproductive stages P: pregnancy, L: lactation, PL: post-lactation.

For the continuous endothermy scenario, Model 10 was the only competitive model (*w_i_* = 0.99, *R*^2^ = 0.86; Supplementary Material, Table S1). We identified 10 informative parameters related to box design, landscape placement, bat abundance, and weather (Supplementary Material, Table S2). DEE_endothermic_ ranged from 10.8 to 37.5 kJ (mean = 19.1 ± 0.09 kJ) and showed substantial variability over time (Fig. 2). DEE_endothermic_ was most variable during pregnancy, with no patterns in differences between landscape placements. In late May, when temperatures warmed and leaf-out occurred in the forests, it was easier to see differences in DEE_endothermic_ by box design (Fig. 2A) and landscape placement (Fig. 2B). Box design had subtle but important impacts on DEE_endothermic_. In the EJW design, mean DEE_endothermic_ was 3.2% lower than in the REF design (Supplementary Material, Table S3). For the CH and WTR designs, mean DEE_endothermic_ was on average 2.1% and 0.5% higher than in the REF design, respectively. Landscape placement had a greater impact, such that in forest placements average DEE_endothermic_ was 9.2% greater than in open placement boxes (Supplementary Material, Table S4). The easterly and westerly sun placements yielded DEE_endothermic_ values very similar to the open placements.

**Figure 3:**
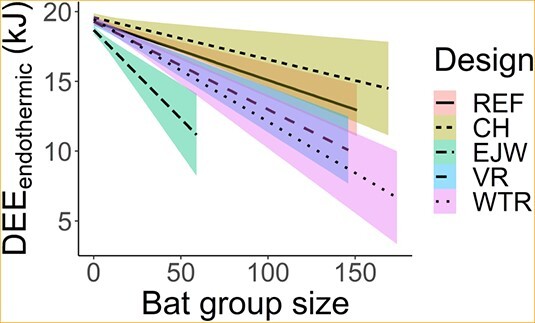
Regression lines and 85% confidence intervals showing the interaction of box design: reference (REF), chimney (CH), external water jacket (EJW), vent removal (VR) and white tile roof (WTR) with total bat group size on daily energy expenditure for continuous endothermy.

Predictably, increasing numbers of bats decreased DEE_endothermic_ for all box designs (Fig. 3). There was a strong decreasing trend in DEE_endothermic_ with increasing bats for the EJW design, but the low maximum emergence count of 59 bats limited our ability to assess the effect of bats for this design. For a group of 50 bats, DEE_endothermic_ was 7.9% lower in the EJW design and 3.9% lower in the WTR than the REF design (Supplementary Material, Table S5). Comparing a roost occupied by 150 bats to an unoccupied roost, predicted DEE_endothermic_ was 29.4% (5 kJ) lower in the WTR design, whereas adding 150 bats to the REF design decreased DEE_endothermic_ by only 12.7% (2.3 kJ). This is due to the WTR design reducing instances of T_roost_ > T_uc_, thus lowering cooling costs at large group sizes.

Increasing maximum daily T_a_ substantially decreased DEE_endothermic_ regardless of placement, and effect sizes were similar for all placements. For instance, for a change in maximum daily T_a_ from 10°C to 30°C, DEE_endothermic_ decreased by 64.4% (17.1 kJ) for forest placements and decreased by 69.5% (16.6 kJ) for open placements. In contrast, DEE_endothermic_ increased substantially as maximum daily wind speed increased, most noticeably in forest placements where T_a_ and solar radiation were lowest. For an increase in maximum daily wind speed from 0 to 7 m/s, DEE_endothermic_ increased by 21.9% (4.7 kJ) at forest placements and by 16.6% (3.1 kJ) at open placements.

### Facultative heterothermy scenario

**Figure 4:**
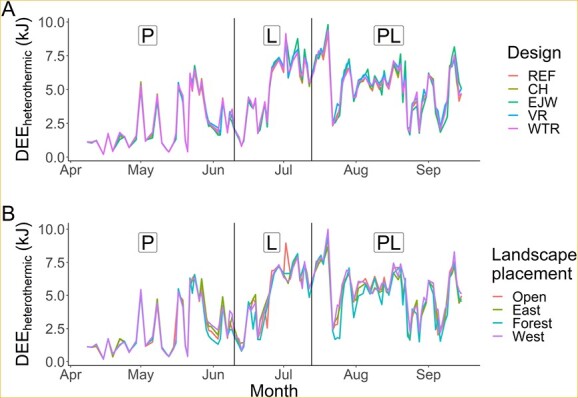
Mean daily energy expenditure (DEE) for facultative heterothermy (DEE_heterothermic_) over the course of the study as a function of (A) box design: reference (REF), chimney (CH), external water jacket (EJW), vent removal (VR), and white tile roof (WTR) and (B) landscape placement: open sun (open), easterly sun (east), westerly sun (west), full shade (forest). Vertical black lines designate reproductive stages P: pregnancy, L: lactation, PL: post-lactation.

Model 14 was the top overall model for the facultative heterothermy scenario (*w_i_* = 0.68, *R*^2^ = 0.91; Supplementary Material, Table S6). Additionally, model 10 was competitive, with ∆AIC*_C_* = 1.5, *w_i_* = 0.32, and *R*^2^ = 0.91. From the 90% confidence set containing models 14 and 10, we identified 10 informative parameters related to box design, landscape position, weather and bat abundance (Supplementary Material, Table S7). DEE_heterothermic_ ranged from 0.2 to 10.4 kJ (mean = 4.1 ± 0.05 kJ), and variability in DEE_heterothermic_ was highest during lactation and post-lactation (Fig. 4). Box design alone did not have a substantial impact on DEE_heterothermic_, which was ~ 4 kJ for all box designs (Supplementary Material, Table S8, Fig 4A), but landscape placement was an important predictor. Compared to the open placements, forest placements decreased DEE_heterothermic_ by 6.9% (Supplementary Material, Table S9, Fig. 4B).

Regardless of box design, increasing numbers of bats increased DEE_heterothermic_, though effects were weak (Supplementary Material, Table S7). Increasing daily maximum T_a_ had a stronger effect by increasing DEE_heterothermic_ in forest placements vs. open placements (Supplementary Material, Table S7). Further, increasing maximum daily wind speed resulted in substantial decreases in DEE_heterothermic_ for all placements. This effect was most prominent for forest placements which had low solar radiation. For instance, for a change in maximum daily wind speed from 0 to 7 m/s, DEE_heterothermic_ decreased in forest placements by 19.7% (0.5 kJ), whereas for open placements DEE_heterothermic_ decreased by 15.9% (0.5 kJ).

### Overheating risk

**Figure 5:**
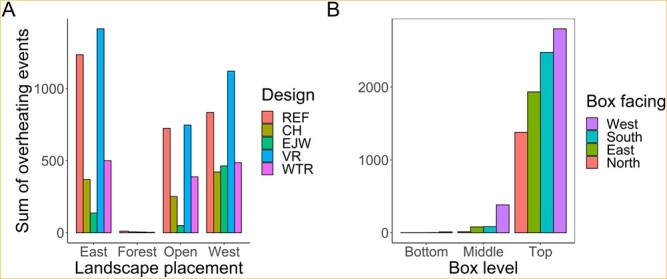
Sum counts of overheating events (> 40°C) at 12 positions within bat boxes by (A) box design: reference (REF), chimney (CH), external water jacket (EJW), vent removal (VR) and white tile roof (WTR), and landscape placement: open sun (open), easterly sun (east), westerly sun (west), full shade (forest), and (B) box level and box face aspect.

In total, boxes recorded 9171 overheating events (i.e. temperatures > 40°C) out of 945 060 total temperature recordings. Most overheating events occurred in the VR and REF designs in solar-exposed locations (66%; Fig. 5A). Further, roosting positions at the top of a bat box overheated most frequently (94%; Fig. 5B). Although no mortality was documented during this study, the maximum temperature recorded by the top position in each design was well above the presumed lethal threshold of 45°C (REF = 53.5°C, CH = 52.5°C, EJW = 48.0°C, VR = 54.5°C and WTR = 51.5°C). Maximum recorded temperatures also varied across landscape placements; only the forest placements remained below the lethal threshold (open = 52.5°C, easterly sun = 52.5°C, westerly sun = 54.5°C, and forest = 43.5°C).

**Figure 6:**
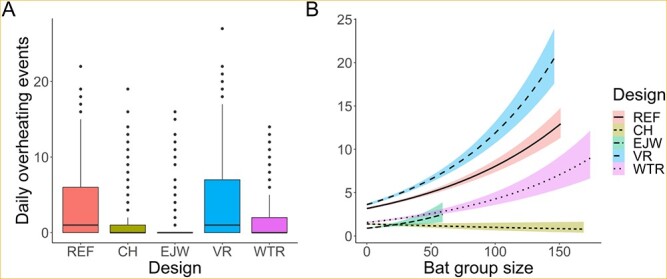
(A) Box and whisker plot of the daily count of overheating events (> 40°C) by box design: reference (REF), chimney (CH), external water jacket (EJW), vent removal (VR) and white tile roof (WTR). (B) Poisson fitted regression lines and 85% confidence intervals displaying the daily count of overheating events by group size (total bats) and box design.

Model 10 was the top model for the overheating analysis (*w_i_* = 1.0, *R*^2^ = 0.61; Supplementary Material, Table S10). From this model, we identified 18 informative parameters related to box design, landscape position, weather and bats (Supplementary Material, Table S11). Design was important; compared to the REF design, the VR design was similar, while the EJW, CH and WTR designs logged substantially fewer daily overheating events (Fig. 6A and Supplementary Material, Table S12). Boxes in easterly and westerly sun placements logged considerably more daily overheating events than boxes in open placements (east: 3.1 ± 0.2, range = 0–27; west: 2.4 ± 0.2, range = 0–22; open: 1.6 ± 0.1, range = 0–22; forest: 0.01 ± 0.01, range = 0–2). Boxes in the open cluster were unused in 2019; overheating should be less likely in the absence of many warm bat bodies. Overheating counts for a given box design varied across placements. Regardless of box design, higher numbers of bats increased overheating risk. This effect was strongest in the VR design (Fig. 6B). Increasing maximum daily temperature increased overheating risk for all three landscape placements tested (Supplementary Material, Table S11); however, easterly and westerly sun placements did not respond as strongly as open placements. On days when T_a_ was ≥30°C, bat boxes logged an average of 5.5 ± 0.2 daily overheating events (range = 0–27 events). Increasing maximum daily wind speed decreased the overheating risk in all placements, most notably in the easterly sun placements (Supplementary Material, Table S11).

## Discussion

### Overview

Our study jointly quantifies the energy benefits and overheating risk of different bat box designs and placements, as well as providing a robust method for assessing the conservation value of bat boxes from two physiological perspectives. We found landscape placement was the most influential, controllable factor altering energy expenditure and overheating risk, but box design, bat group size, and weather were also important factors. For reproductively active bats maintaining endothermy, solar-exposed landscape placements resulted in the lowest DEE_endothermic,_ and the EJW box (with greater thermal mass) promoted further reductions in DEE_endothermic_. For facultatively heterothermic bats, forest placements resulted in the lowest DEE_heterothermic_ and virtually eliminated overheating events. At the same time, our study affirms overheating risk is higher in solar-exposed locations and risk is further increased by box designs with low surface reflectance, low thermal mass or poor ventilation (Griffiths *et al.,* 2017; Martin Bideguren *et al.,* 2019; Rowland *et al.*, 2017; Tillman *et al.*, 2021). Three box designs (CH, EJW and WTR) reduced overheating risk compared to the REF design, but only one (EJW) simultaneously decreased endothermic energy expenditure. These observations suggest there need not be a tradeoff in reducing box overheating risk and providing a microclimate suitable for reproductive bats.

### Landscape

Landscape placement is a key consideration for practitioners when choosing to deploy bat boxes. For instance, both high solar exposure and aspects facing the sun are qualities of bat boxes facilitating maternity colony formation (Brittingham and Williams, 2000; Kerth *et al.*, 2001) and can increase bat box uptake (Whitaker *et al.*, 2006). For a continuously endothermic individual, easterly sun, westerly sun and open sun placements yielded the lowest DEE. This validates the assertion that female bats choose solar-exposed roosts to reduce the energy costs of normothermia (Callahan *et al.*, 1997; Vonhof and Barclay, 1996), thus facilitating the avoidance of torpor use and the negative effects of low body temperature on reproduction (e.g. Hoying and Kunz, 1998; Wilde *et al.*, 1999; but see Bergeson *et al.*, 2021). In contrast, forest box placements minimized DEE for heterothermic bats. Less solar exposure of this landscape placement likely tempered warm roosting positions, thus facilitating deeper torpor bouts and, hence, greater energy savings. While reproductive bats gain both energetic and reproductive benefits from solar-exposed roosts, our study and others indicate high solar exposure and orientations facing the sun increases overheating risk (Griffiths *et al.,* 2017; Martin Bideguren *et al.,* 2019). The effects of landscape placement can be mitigated (to some extent) with careful box design selection; however, overheating risk must be minimized while still providing warm microclimates to facilitate pup growth (Crawford and O’Keefe, 2021a).

### Box design

While past bat box bioenergetic research has focused on the impacts of artificial roost colour and heating on energy expenditure (e.g. Doty *et al.*, 2016; Wilcox and Willis, 2016), few studies have specifically assessed overheating risk posed by varying box designs (but see, Flaquer *et al.,* 2014; Griffiths *et al.,* 2017; Martin Bideguren *et al.,* 2019). We expand on prior work by interactively investigating the effects of a box’s thermal mass, ventilation, and enhanced surface reflectance on DEE and overheating risk. By roosting in the EJW design (which has higher thermal mass than REF), bats can reduce DEE_endothermic_ and simultaneously experience a lower risk of overheating compared to the REF design. Similarly, nest boxes insulated with polystyrene foam have lower thermal conductance and greater buffering capacity, thus retaining more heat at night (Larson *et al.*, 2018). Increasing bat box thermal mass should decrease metabolic costs for normothermic endotherms. In our study, boxes that decreased overheating events through higher surface reflectance (WTR) or enhanced ventilation (CH) also increased DEE_endothermic_. Thus, there is a tradeoff between heat stress risk and energetic benefits in some instances.

The number of bats occupying a bat box can alter temperature and energetic savings (Pretzlaff *et al.*, 2010; Willis and Brigham, 2007), but this effect varies with bat box design. For example, with increasing bat group size, the WTR and EJW designs had stronger decreasing trends in DEE_endothermic_ compared to REF. Similarly, artificial boxes insulated with polystyrene and occupied by individual great tits (*Parus major*) were significantly warmer than T_a_, whereas occupation by a single bird did not significantly elevate box temperature in uninsulated nest boxes (Veľký *et al.*, 2010). Our work also highlights the value of altering thermal mass and thermal conductance in artificial roosts to reduce overheating risk and potentially enhance social thermoregulation at large bat group sizes.

Contrary to our prediction, box design was not an important factor for DEE_heterothermic_. Rocket boxes support vertical temperature gradients up to 10°C (Hoeh *et al.*, 2018), offer multiple aspect options (i.e. north, south, east and west faces), and ~1 m of vertical space; these bat box traits should allow for bats to find temperatures suitable for torpor use in all our roost designs, assuming colony size does not hinder movement. We found increasing bat group size increased overall energy expenditure because of the concomitant increase in T_roost_ and because metabolic rate increases with temperature for torpid bats (Geiser and Brigham, 2000; Willis *et al.,* 2005a). Only in the CH design did we see that increasing group size did not impact energy expenditure, likely due to excess body heat generated by bats being vented out the chimney and, thus, keeping temperatures cooler for torpid bats.

### Conservation implications

While our data are specific to bats, the insights derived from our analysis are broadly applicable to a variety of nest-box using mammals. Our work highlights several key considerations that may improve the success of bat boxes when deployed for the conservation of imperilled bat species. Landscape placement in conjunction with bat box design and colour are key aspects of mitigating overheating risk to bats and creating conditions conducive to endothermy. Deploying a design with inadequate features (e.g. poor ventilation, low surface reflectance, small size) in a solar-exposed location is likely to increase the risk of heat stress to roosting bats. When deploying bat boxes for maternal bat populations, practitioners should consider the use of box designs that increase thermal mass (EJW in this study) to buffer against overheating events while simultaneously decreasing DEE_endothermic_. We recommend further experiments altering thermal mass, thermal conductance and surface reflectance to improve upon current bat box designs, as these modifications will be critical to buffering the immediate effects of overheating and long-term effects of a warming climate (Larson *et al.*, 2018).

Promoting warmer microclimates in roosts during the spring and summer could be critical to improve survival rates of bats impacted by diseases, like white-nose syndrome (Wilcox and Willis, 2016). For example, little brown bats maintain higher body temperatures when rapidly healing from white-nose syndrome during the spring (Fuller *et al.,* 2020). Supplying afflicted bats with roosts that reduce the overall costs of maintaining higher body temperatures could promote recovery. Further, increasing the energy savings of bats during the summer by catering to both heterothermic and endothermic bats could enhance overwinter survival. For instance, little brown bats going into hibernation with larger fat stores have a higher probability of surviving hibernation (Cheng *et al.,* 2018). Clearly, additional work is needed to improve upon current bat box designs to increase energy savings while simultaneously reducing overheating events (Crawford and O’Keefe, 2021a). While no mortality was documented during the study period (2019), we observed mortality (presumably from overheating) at boxes in both study sites during the summer of 2020 (11 bats in Indiana and 4 bats in Kentucky) and in Kentucky in 2021 (2 bats). We urge practitioners to recognize the importance of bat box design, material, colour and placement, and to carefully study microclimates of novel artificial roosts before provisioning artificial roosts to bats (Crawford and O’Keefe, 2021b).

While artificial roost microclimates are relatively easy to study and manipulate, we lack data on how artificial roost microclimates compare to natural tree roost microclimates. Many tree roosts (e.g. hollows, cavities and bark) buffer against temperature extremes, such that they are cooler than outside air temperatures during the day and warmer at night (Lacki *et al.*, 2013; O’Connell and Keppel, 2016; Rowland *et al.*, 2017; Sedgeley, 2001). Most solar-exposed artificial roosts exceed ambient temperature during the day (Griffiths *et al.,* 2017; Martin Bideguren *et al.,* 2019; Tillman *et al.*, 2021, this study), sometimes by > 25°C (Hoeh *et al.*, 2018), and fall to ambient temperature within a few hours of sunset (Kerth *et al.*, 2001; Lourenço and Palmeirim, 2004; Tillman*et al.*, 2021). We recommend future work to compare the microclimates of both natural and artificial roosts from both physiological and ecological perspectives to improve our understanding of artificial roost use.

## Funding

This work was supported by the Graduate School of Eastern Kentucky University, the University-Funded Scholarship Program at Eastern Kentucky University (award no.19-215), the Imperiled Bat Conservation Fund administered by the Kentucky Natural Lands Trust, the Joint Fire Sciences Program (award no. 14-1-05-22), the United States Fish and Wildlife Service (award no. F20AO00273) and the Kentucky Department of Fish and Wildlife Resources (award no. PON66018000010241).

## Author Contributions

Reed D. Crawford: conceptualization, data collection, analysis, writing and revision. Luke E. Dodd: conceptualization, data collection, analysis, writing and revision. Francis E. Tillman: conceptualization, data collection, and revision. Joy M O’Keefe: conceptualization, data collection, analysis, writing, and revision.

## Data Availability

The data are available in the Illinois Data Bank at the University of Illinois at Urbana-Champaign. (https://doi.org/10.13012/B2IDB-3592866_V1).

## Supplementary Material

Web_Material_coac027
