# Supplementary material for: Evaluating bat boxes: design and placement alter bioenergetic costs and overheating risk
Source: Conserv Physiol. 2022 Apr 25;10(1):coac027. doi: 10.1093/conphys/coac027 (PMC9041549; doi:10.1093/conphys/coac027)
Supplement: Web_Material_coac027 [file web_material_coac027.docx]

***Conservation Physiology***

**Supplemental Material**

**Evaluating bat boxes: Design and placement alter bioenergetic costs and overheating risk**

**Crawford, R. D., L. E. Dodd., F. E. Tillman., and J. M. O’Keefe**

*Continuous endothermy analysis supplementary tables*

Table S1: AIC*_C_* model selection results for the continuous endothermy analysis.

| Model | ∆AIC*_C_* | *df* | *w_i_* |
| --- | --- | --- | --- |
| m10 | 0.0 | 34 | 0.99 |
| m9 | 9.2 | 36 | 0.01 |
| m14 | 70.4 | 29 | <0.001 |
| m13 | 76.1 | 31 | <0.001 |
| m8 | 239.9 | 30 | <0.001 |
| m7 | 250.6 | 31 | <0.001 |
| m12 | 316.2 | 25 | <0.001 |
| m11 | 322.3 | 26 | <0.001 |
| m5 | 4795.8 | 14 | <0.001 |
| m6 | 4817.3 | 26 | <0.001 |
| m3 | 4871.8 | 5 | <0.001 |
| m4 | 4886.1 | 21 | <0.001 |
| m2 | 4936.8 | 6 | <0.001 |
| null | 4940.2 | 2 | <0.001 |

Table S2: Parameters, parameter estimates, and lower and upper 85% confidence intervals for the top ranked continuous endothermy analysis model (model 10). Informative parameters in bold.

|  |  | 85% CI | |
| --- | --- | --- | --- |
| Parameter | Estimate | lower | upper |
| (Intercept) | 39.66252 | 38.85251 | 40.47253 |
| **DesignCH** | **0.32422** | **0.01551** | **0.63292** |
| **DesignEJW** | **-0.46618** | **-0.77431** | **-0.15805** |
| DesignVR | -0.13108 | -0.43921 | 0.17706 |
| **DesignWTR** | **0.33819** | **0.03006** | **0.64633** |
| PlacementEast | 0.45925 | -0.59353 | 1.51204 |
| **PlacementForest** | **2.58709** | **1.51077** | **3.66341** |
| PlacementWest | 0.60459 | -0.43510 | 1.64428 |
| **Total.Bats** | **-0.01554** | **-0.02085** | **-0.01024** |
| **Max_Temp** | **-0.83420** | **-0.85722** | **-0.81117** |
| **Max_Wind** | **0.45095** | **0.38001** | **0.52188** |
| DesignCH:PlacementEast | 0.16021 | -0.29355 | 0.61397 |
| DesignEJW:PlacementEast | -0.18910 | -0.65999 | 0.28179 |
| DesignVR:PlacementEast | -0.14599 | -0.61457 | 0.32259 |
| DesignWTR:PlacementEast | -0.21472 | -0.66543 | 0.23600 |
| DesignCH:PlacementForest | 0.04332 | -0.40241 | 0.48906 |
| DesignEJW:PlacementForest | -0.26040 | -0.70527 | 0.18446 |
| DesignVR:PlacementForest | 0.15360 | -0.29978 | 0.60697 |
| DesignWTR:PlacementForest | -0.19117 | -0.64051 | 0.25816 |
| DesignCH:PlacementWest | 0.13738 | -0.30521 | 0.57998 |
| **DesignEJW:PlacementWest** | **-0.61292** | **-1.05501** | **-0.17083** |
| DesignVR:PlacementWest | -0.09811 | -0.53744 | 0.34122 |
| DesignWTR:PlacementWest | -0.30263 | -0.74494 | 0.13967 |
| DesignCH:Total.Bats | 0.00413 | -0.00513 | 0.01338 |
| DesignEJW:Total.Bats | -0.01451 | -0.03458 | 0.00556 |
| DesignVR:Total.Bats | -0.00442 | -0.01334 | 0.00449 |
| **DesignWTR:Total.Bats** | **-0.01737** | **-0.02662** | **-0.00812** |
| PlacementEast:Max_Temp | 0.02262 | -0.00855 | 0.05380 |
| PlacementForest:Max_Temp | -0.02015 | -0.05405 | 0.01375 |
| PlacementWest:Max_Temp | 0.02716 | -0.00360 | 0.05793 |
| PlacementEast:Max_Wind | -0.03401 | -0.14342 | 0.07540 |
| **PlacementForest:Max_Wind** | **0.22829** | **0.08168** | **0.37490** |
| PlacementWest:Max_Wind | -0.07634 | -0.17432 | 0.02165 |

Table S3: Daily energy expenditure means and ranges by bat box design for the continuous endothermy analysis**.**

| Design | Mean DEE ± SE (kJ) | Range (kJ) |
| --- | --- | --- |
| REF | 19.1 ± 0.20 | 12.2–37.3 |
| CH | 19.5 ± 0.21 | 12.7–37.3 |
| EJW | 18.5 ± 0.22 | 11.8–36.6 |
| VR | 19.0 ± 0.21 | 12.5-37.0 |
| WTR | 19.2 ± 0.21 | 10.8–37.5 |

Table S4: Daily energy expenditure means and ranges by placement for the continuous endothermy analysis**.**

| Placement | Mean DEE ± SE (kJ) | Range (kJ) |
| --- | --- | --- |
| Open | 18.7 ± 0.19 | 11.8–37.5 |
| East | 18.5 ± 0.20 | 11.8–37.3 |
| West | 18.6 ± 0.18 | 10.8–36.6 |
| Forest | 20.5 ± 0.17 | 13.1–37.1 |

Table S5: Predicted daily energy expenditure means and ranges for bat box designs occupied by 50 bats for the continuous endothermy analysis**.** Predictions generated through ‘emmeans’.

| Design | Group Size (Bats) | Predicted Mean DEE ± SE (kJ) |
| --- | --- | --- |
| REF | 50 | 18.5 ± 0.19 |
| CH | 50 | 19.1 ± 0.26 |
| EJW | 50 | 17.1 ± 0.66 |
| VR | 50 | 18.1 ± 0.24 |
| WTR | 50 | 17.8 ± 0.26 |

*Facultative heterothermy analysis supplementary tables*

Table S6: AIC*_C_* model selection results for the facultative heterothermy analysis.

| Model | ∆AIC*_C_* | *df* | *w_i_* |
| --- | --- | --- | --- |
| m14 | 0.0 | 29 | 0.68 |
| m10 | 1.5 | 34 | 0.32 |
| m13 | 91.9 | 31 | <0.001 |
| m9 | 94.6 | 36 | <0.001 |
| m12 | 98.1 | 25 | <0.001 |
| m8 | 100.0 | 30 | <0.001 |
| m11 | 153.0 | 26 | <0.001 |
| m7 | 156.5 | 31 | <0.001 |
| m5 | 5999.1 | 14 | <0.001 |
| m3 | 6019.9 | 5 | <0.001 |
| m6 | 6022.0 | 26 | <0.001 |
| null | 6024.7 | 2 | <0.001 |
| m2 | 6031.4 | 6 | <0.001 |
| m4 | 6049.3 | 21 | <0.001 |

Table S7: Parameters, model averaged parameter estimates, and lower and upper 85% confidence intervals for the facultative heterothermy analysis (models 14 and 10). Informative parameters in bold.

|  |  | 85% CI | |
| --- | --- | --- | --- |
| Parameter | Estimate | lower | upper |
| (Intercept) | -3.07920 | -3.19412 | -2.96429 |
| DesignCH | -0.00362 | -0.04741 | 0.04018 |
| DesignEJW | -0.01824 | -0.06195 | 0.02548 |
| DesignVR | 0.00723 | -0.03648 | 0.05094 |
| DesignWTR | 0.00525 | -0.03846 | 0.04896 |
| PlacementEast | 0.08482 | -0.06438 | 0.23402 |
| **PlacementForest** | **-0.28698** | **-0.43967** | **-0.13429** |
| PlacementWest | 0.13399 | -0.01340 | 0.28138 |
| **Max_Temp** | **0.15901** | **0.15574** | **0.16228** |
| **Max_Wind** | **-0.02266** | **-0.03272** | **-0.01259** |
| PlacementEast:DesignCH | 0.00592 | -0.05831 | 0.07015 |
| PlacementEast:DesignEJW | -0.01138 | -0.07717 | 0.05441 |
| PlacementEast:DesignVR | 0.01177 | -0.05273 | 0.07627 |
| PlacementEast:DesignWTR | -0.00582 | -0.06964 | 0.05800 |
| PlacementForest:DesignCH | 0.02214 | -0.04110 | 0.08537 |
| PlacementForest:DesignEJW | -0.01585 | -0.07897 | 0.04726 |
| **PlacementForest:DesignVR** | **0.06532** | **0.00100** | **0.12964** |
| PlacementForest:DesignWTR | 0.01823 | -0.04551 | 0.08198 |
| PlacementWest:DesignCH | -0.00846 | -0.07112 | 0.05419 |
| PlacementWest:DesignEJW | 0.03584 | -0.02672 | 0.09839 |
| PlacementWest:DesignVR | 0.03710 | -0.02517 | 0.09937 |
| PlacementWest:DesignWTR | 0.03375 | -0.02885 | 0.09635 |
| PlacementEast:Max_Temp | -0.00409 | -0.00853 | 0.00035 |
| **PlacementForest:Max_Temp** | **0.01378** | **0.00897** | **0.01859** |
| **PlacementWest:Max_Temp** | **-0.00667** | **-0.01103** | **-0.00231** |
| PlacementEast:Max_Wind | -0.01211 | -0.02762 | 0.00339 |
| **PlacementForest:Max_Wind** | **-0.07571** | **-0.09651** | **-0.05491** |
| PlacementWest:Max_Wind | -0.00582 | -0.01971 | 0.00807 |
| **Total.Bats** | **0.00078** | **0.00003** | **0.00154** |
| **DesignCH:Total.Bats** | **-0.00138** | **-0.00269** | **-0.00007** |
| DesignEJW:Total.Bats | 0.00038 | -0.00247 | 0.00322 |
| DesignVR:Total.Bats | 0.00077 | -0.00049 | 0.00204 |
| **DesignWTR:Total.Bats** | **-0.00137** | **-0.00268** | **-0.00006** |

Table S8: Daily energy expenditure means and ranges by bat box design for the facultative heterothermy analysis**.**

| Design | Mean DEE ± SE (kJ) | Range (kJ) |
| --- | --- | --- |
| REF | 4.0 ± 0.10 | 0.2–10.0 |
| CH | 4.1 ± 0.10 | 0.2–10.0 |
| EJW | 4.0 ± 0.11 | 0.2–10.4 |
| VR | 4.1 ± 0.11 | 0.2–10.0 |
| WTR | 4.1 ± 0.10 | 0.2–10.0 |

Table S9: Daily energy expenditure means and ranges by placement for the facultative heterothermy analysis**.**

| Placement | Mean DEE ± SE (kJ) | Range (kJ) |
| --- | --- | --- |
| Open | 4.1 ± 0.09 | 0.2–9.9 |
| East | 4.2 ± 0.09 | 0.2–9.4 |
| West | 4.3 ± 0.09 | 0.2–10.0 |
| Forest | 3.8 ± 0.10 | 0.2–10.4 |

*Overheating analysis*

Table S10: AIC*_C_* model selection results for the overheating analysis.

| Model | ∆AIC*_C_* | *df* | *w_i_* |
| --- | --- | --- | --- |
| m10 | 0 | 26 | 1 |
| m9 | 94.7 | 30 | <0.001 |
| m14 | 105.4 | 21 | <0.001 |
| m13 | 216.7 | 25 | <0.001 |
| m8 | 834.1 | 23 | <0.001 |
| m7 | 848.7 | 25 | <0.001 |
| m12 | 930.8 | 18 | <0.001 |
| m11 | 950.2 | 20 | <0.001 |
| m6 | 6327.1 | 20 | <0.001 |
| m5 | 6524 | 12 | <0.001 |
| m4 | 6539.5 | 15 | <0.001 |
| m2 | 7039.1 | 5 | <0.001 |
| m3 | 7994.9 | 3 | <0.001 |
| null | 8298.3 | 1 | <0.001 |

Table S11: Parameters, parameter estimates, and lower and upper 85% confidence intervals for the top ranked overheating analysis model (model 10). Informative parameters in bold.

|  |  | 85% CI | |
| --- | --- | --- | --- |
| Parameter | Estimate | lower | upper |
| (Intercept) | -11.25565 | -11.95318 | -10.56532 |
| **DesignCH** | **-1.08132** | **-1.23456** | **-0.93187** |
| **DesignEJW** | **-2.79506** | **-3.12684** | **-2.49289** |
| DesignVR | 0.05407 | -0.05178 | 0.16002 |
| **DesignWTR** | **-0.62341** | **-0.75272** | **-0.49577** |
| **PlacementEast** | **3.19578** | **2.36096** | **4.03392** |
| **PlacementWest** | **0.97020** | **0.07366** | **1.86724** |
| **Total.Bats** | **0.00512** | **0.00400** | **0.00622** |
| **Max_Temp** | **0.46143** | **0.44009** | **0.48301** |
| **Max_Wind** | **-0.61602** | **-0.67306** | **-0.55895** |
| DesignCH:PlacementEast | 0.11095 | -0.08617 | 0.30930 |
| **DesignEJW:PlacementEast** | **1.21287** | **0.85957** | **1.58697** |
| DesignVR:PlacementEast | -0.06299 | -0.20712 | 0.08105 |
| **DesignWTR:PlacementEast** | **-0.20754** | **-0.37762** | **-0.03700** |
| **DesignCH:PlacementWest** | **0.57061** | **0.37670** | **0.76636** |
| **DesignEJW:PlacementWest** | **2.32547** | **1.99816** | **2.67835** |
| **DesignVR:PlacementWest** | **0.22771** | **0.08348** | **0.37214** |
| DesignWTR:PlacementWest | -0.05909 | -0.24511 | 0.12678 |
| **DesignCH:Total.Bats** | **-0.01523** | **-0.02136** | **-0.01004** |
| DesignEJW:Total.Bats | -0.00847 | -0.01857 | 0.00056 |
| **DesignVR:Total.Bats** | **0.00363** | **0.00172** | **0.00552** |
| DesignWTR:Total.Bats | -0.00030 | -0.00282 | 0.00212 |
| **PlacementEast:Max_Temp** | **-0.13156** | **-0.15743** | **-0.10582** |
| **PlacementWest:Max_Temp** | **-0.05067** | **-0.07881** | **-0.02256** |
| **PlacementEast:Max_Wind** | **0.38045** | **0.31028** | **0.45045** |
| PlacementWest:Max_Wind | 0.00348 | -0.06730 | 0.07410 |

Table S12: Daily overheating event means and ranges by bat box design for the overheating analysis**.**

| Design | Mean ± SE | Range |
| --- | --- | --- |
| REF | 3.5 ± 0.26 | 0–22 |
| CH | 1.4 ± 0.16 | 0–19 |
| EJW | 0.9 ± 0.14 | 0–16 |
| VR | 4.1 ± 0.30 | 0–27 |
| WTR | 1.7 ± 0.14 | 0–14 |
